# *In Vitro* Structural and Functional Characterization of the Small Heat Shock Proteins (sHSP) of the Cyanophage S-ShM2 and Its Host, *Synechococcus sp*. WH7803

**DOI:** 10.1371/journal.pone.0162233

**Published:** 2016-09-19

**Authors:** Maxime Bourrelle-Langlois, Geneviève Morrow, Stéphanie Finet, Robert M. Tanguay

**Affiliations:** 1 Laboratoire de Biologie Cellulaire et Moléculaire, Institut de Biologie Intégrative et des Systémes (IBIS) and PROTEO, Département de biologie moléculaire, biochimie médicale et pathologie, Faculté de Médecine, Québec, Canada; 2 IMPMC UMR7590, CNRS/Sorbonne-Universités, UPMC/IRD/MNHN Paris 6, Paris, France; Universite de Geneve, SWITZERLAND

## Abstract

We previously reported the *in silico* characterization of *Synechococcus* sp. phage 18 kDa small heat shock protein (HspSP-ShM2). This small heat shock protein (sHSP) contains a highly conserved core alpha crystalline domain of 92 amino acids and relatively short N- and C-terminal arms, the later containing the classical C-terminal anchoring module motif (L-X-I/L/V). Here we establish the oligomeric profile of HspSP-ShM2 and its structural dynamics under *in vitro* experimental conditions using size exclusion chromatography (SEC/FPLC), gradient native gels electrophoresis and dynamic light scattering (DLS). Under native conditions, HspSP-ShM2 displays the ability to form large oligomers and shows a polydisperse profile. At higher temperatures, it shows extensive structural dynamics and undergoes conformational changes through an increased of subunit rearrangement and formation of sub-oligomeric species. We also demonstrate its capacity to prevent the aggregation of citrate synthase, malate dehydrogenase and luciferase under heat shock conditions through the formation of stable and soluble hetero-oligomeric complexes (sHSP:substrate). In contrast, the host cyanobacteria *Synechococcus* sp. WH7803 15 kDa sHSP (HspS-WH7803) aggregates when in the same conditions as HspSP-ShM2. However, its solubility can be maintained in the presence of non-ionic detergent Triton^™^X-100 and forms an oligomeric structure estimated to be between dimer and tetramer but exhibits no apparent inducible structural dynamics neither chaperon-like activity in all the assays and molar ratios tested. SEC/FPLC and thermal aggregation prevention assays results indicate no formation of hetero-oligomeric complex or functional interactions between both sHSPs. Taken together these *in vitro* results portray the phage HspSP-ShM2 as a classical sHSP and suggest that it may be functional at the *in vivo* level while behaving differently than its host amphitropic sHSP.

## Introduction

The small heat shock proteins (sHSPs) are ubiquitous ATP-independent molecular chaperones participating in many cellular processes but mainly characterized for their role in proteostasis [1–3]. They are composed of highly variable N- (NTR) and C-terminal (CTR) regions flanking the conserved ~ 90 amino acids α-crystallin domain (ACD) and have an average molecular masses of 12-kDa to 42-kDa [4, 5]. sHSPs generally form oligomers ranging from 12 to more than 32 subunits when at equilibrium. They are either present in one main oligomeric form (monodispersity; [6, 7]) or in a variety of oligomeric forms with different abundance (polydispersity; [8–11]) reflecting a high dynamism in quaternary structure conformation as a result of rapid and extensive subunit exchange between oligomers. Under stress conditions such as heat, sHSPs chaperone activity prevents irreversible aggregation maintaining protein clients solubility by binding hydrophobic exposed surfaces while in process of stress-induced denaturation [12]. *In vitro* [13–15] and *in vivo* [16–18] experimentations suggest a promiscuous nature of chaperone activity specificity for sHSPs. Upon protein client binding there is formation of ordered and soluble sHSPs:clients complexes of higher molecular mass than the client free oligomers [19, 20]. Then the ATP-dependent chaperone machinery Hsp70/Hsp40 (DnaK/DnaJ), Hsp60 (GrpE) and Hsp100 (CIpB) release and actively refold partially denatured protein client [21–23]. In cyanobacteria, amphitropic sHSPs (Hsp16.6, Hsp17 or HspA) also localize and associate to thylakoid membranes via membrane proteins or lipids. By increasing protein/lipid ratio thereby modulating fluidity and microviscosity, amphitropic sHSPs allow conservation of membrane integrity under heat stress conditions [24–28].

In 2010 Sullivan and colleagues [29] were the first to report the presence of a *shsp* gene in marine viruses. This was followed by an *in silico* phylogenetic analysis of sHSPs in cyanobacteria and their phages and characterization of monomer tertiary structure [30]. Only cyanophages infecting picophytoplankton *Synechococcus* spp. and *Prochlorococcus* spp. have a *shsp* gene. Phages sHSP sequence analysis showed a conserved ~92 amino acids ACD, shorter than average but relatively conserved N-terminal region (NTR) and C-terminal region (CTR) with L-X-I/L/V C-terminal anchoring module (CAM) motif and other sequence characteristics of bacterial sHSPs. Cyanobacteria sHSPs sequences are related to those of plants. These phylogenetic relationships point toward a horizontal gene transfer event between cyanophages and cyanobacteria that happened million years ago. This implies that cyanophage *shsp* gene has evolved, at some point, independently and differently from its actual hosts cyanobacteria and, yet, co-evolved with cyanobacteria hosts while in pseudo- or lysogenic phase. Furthermore, these cyanophages, according to species, also possess other cyanobacteria related genes [29] mainly involved in carbon metabolism, stress tolerance and photosynthesis [31–34]. Co-transcription with viral capsid gene driven by separate promoter sequences as proposed for cyanobacterial homologous *psbA* (photosystem II core D1 protein) and *hli* (high light inducible protein) cyanophage encoding genes [35, 36] indicates lytic entering phase specific expression. Such expression of phage « photosynthesis » genes ensure the repair cycle of photosynthetic apparatus and energy production while undergoing virus production for lysis.

In order to establish the functional and structural properties of cyanophages sHSP, we have selected the *shsp* gene sequence from cyanophage S-ShM2 (HspSP-ShM2) and the one from its cyanobacterial host *Synechococcus* sp. WH7803 (HspS-WH7803) for *in vitro* protein production and purification. The aim of this study was to characterize the structure and function of the sHSP from the cyanophage S-ShM2 to compare it to the profile of sHSP from the host cyanobacteria as well as to explore the possibility of an interaction or functional cooperation between both sHSPs.

## Materials and Methods

### Genes cloning, protein production and purification

HspSP-ShM2 coding gene (GI: 310003214, base pairs 140422 to 140919 and accession number: GU071096) from cyanophage S-ShM2 genome and HspS-WH7803 coding gene (GI: 147846875, base pairs 2233963 to 2234373 and accession number: CT971583) from *Synechococcus* sp. WH7803 genome were synthetized by GenScript (Piscataway, NJ). Genes were amplified by PCR for subsequent insertion into pETHSUK vector (generously provided by Dr Stephen D. Weeks, KU Leuven, Belgium) via Gibson assembly^®^ (New England Biolabs, Cat No: E5510S). Primers (Invitrogen^™^, Carlsbad, CA) used for *hspSP-ShM2-pETHSUK* and *hspS-WH7803-pETHSUK* PCR amplification and cloning are listed in Table 1. Amplifications were carried out by mixing 100 ng of *shsp* DNA template, 0.5 μl of 10 mM dNTP, 2.5 μl of each corresponding primers.

**Table 1:** Forward and reverse primers for *hspSP-ShM2* and *hspS-WH7803* PCR amplification and cloning by virtue of Gibson Assembly®.

Insert−vector	Sense	Primer sequence (5'-3')
*hspSP-ShM2*−*pETHSUK*	Fwd^a^	GAACAGATTGGTGGTACCATGACTGGACTGAGAAAGTTC
	Rev^b^	GGGCTTTGTTAGCAGATTATAGTGTGTCGCTGGATGAC
*hspS-WH7803*−*pETHSUK*	Fwd^a^	GAACAGATTGGTGGTACCATGATCACCCTTCGTCAATCACCATTCGATCTC
	Rev^b^	GGGCTTTGTTAGCAGATTAGGCGTCGACGGCCACCGT

^a^Forward primer

^b^Reverse primer

(5 μM), 5 μl of Q5^®^ 5X Reaction Buffer (New England Biolabs, Cat. No: B9027S) and 0.25 μl of Q5^®^ High-Fidelity DNA Polymerase (New England Biolabs, Cat. No: M0491S). Mixtures were incubated 30 s at 98°C followed by 30 cycles of 10 s at 98°C, 30 s at 62°C, 30 s at 72°C and a final elongation period of 2 min at 72°C. Amplified *shsps* DNA were cloned into pETHSUK vector [37] as described in the Gibson Assembly^®^ protocol. Prior to the insertion procedure, pETHSUK vector was linearized using KpnI and HindIII restriction enzymes. Both s*hsp*-pETHSUK DNA constructions were verified by DNA profile upon restriction enzyme digestion on agarose gel and then by DNA sequencing (Genomic Analysis Platform, Institut de Biologie Intégrative et des Systèmes (IBIS), Université Laval, Québec, Canada).

For protein production each construction was transformed into competent *E*.*coli* BL21 (DE3) (New England Biolabs, Massachusetts, USA). Transformed bacteria were incubated in LB broth Miller (EMD Chemicals Inc., Darmstadt, Germany) at 37°C until reaching midlog phase of bacterial growth (OD_600_ of 0.6). From there, induction of recombinant sHSPs transcription was induced with 0.3 mM isopropyl-β-D-thiogalactoside (IPTG) (Roche Applied Science, Mannheim, Germany) for 5 hours at 30°C. Then bacteria were harvested by centrifugation at 4,000 g for 10 min at 4°C and conserved at -80°C.

Bacterial pellets were resuspended in 0.01 M Tris-Hcl pH 7.9, 0.1 M sodium phosphate, 0.01 mg/ml of DNase and 0.004 M of phenylmethanesulfonyl fluoride (PMSF) and lysed with FRENCH^®^ press (Aminco FRENCH^®^ pressure cell press). Bacteria lysates containing His6-SUMO-HspSP-ShM2 were centrifuged at 4,000 g for 5 min at 4°C and supernatant was harvested. The supernatant was centrifuged a second time at 30,000 g for 30 min at 4°C and supernatant was used for subsequent affinity chromatography. His6-SUMO-HspS-WH7803 lysates, on the other hand, were centrifuged at 4,000 g for 5 min and 30 min at 100,000 g, both at 4°C, for extensive lipid removal. For affinity chromatography in denaturing conditions, supernatants were pre-incubated in 8 M urea and then loaded onto a column filled with 1.5 ml of charged nickel agarose beads (Ni-NTA) (Qiagen, Hilden, Germany) and equilibrated with denaturing binding buffer (8 M urea, 0.1 M sodium phosphate, 0.01 M Tris-HCl pH 8.0) for ionic interaction with His6 tag. The nickel column was then washed with denaturing binding buffer at pH 6.3 and His6-SUMO-HSPs elution was performed with denaturing binding buffer at pH 4.5. Eluted sHSPs were submitted to sequential dialysis in TEN buffer (50 mM Tris-HCl pH 8.0, 0.1 mM EDTA and 150 mM NaCl) with decreasing concentration of urea until complete removal. Proteins were concentrated using Amicon^®^ Ultra-2ml 10-kDa cut off centrifugal filter units (Merck Millipore, Ireland) and protein concentration was measured with the Bradford method (Bio-Rad protein Assay, Bio-Rad, California, USA). The His6-SUMO tag was then removed by digestion with recombinant His6-SUMO-1 hydrolase at 30°C for 1 h. In the case of digested HspSP-ShM2, a re-incubation in denaturing binding buffer followed by an affinity chromatography as previously described enabled us to harvest the sHSP in the flow-through fraction while His6-SUMO tag and His6-SUMO-1 hydrolase remained bound to the beads. HspS-WH7803 on the other hand aggregates after tag digestion. A centrifugation at 16,000 g for 15 min at room temperature allowed to pellet the sHSP aggregates and to get rid of the His6-SUMO tag and His6-SUMO-1 hydrolase in the soluble phase. The pellet was then resuspended in TEN buffer containing 0.23 mM TritonX-100^™^, the critical micelle concentration (CMC) of TritonX-100^™^, to enhance HspS-WH7803 solubilization through hydrophobic interactions with the non-ionic detergent.

### Size Exclusion Chromatography (SEC)

SEC analyses were performed using a Superdex 200 increase 10/300 GL column (GE Healthcare Life Sciences, Björkgatan, Sweden) and an ÄKTA pure 25L (GE Healthcare Life Sciences, Björkgatan, Sweden) system. For oligomeric profile investigation, the column was equilibrated with ~ 35 ml of TEN buffer for HspSP-ShM2 alone and TEN supplemented with 0.23 mM or 0.12 mM TritonX-100^™^ for HspS-WH7803 alone and mixed with HspSP-ShM2. All samples were centrifuged at room temperature for 15 min at 13,000 rpm to remove aggregates and the corresponding supernatants were loaded onto the column. Proteins were eluted at a flow rate of 0.5 ml/min at room temperature. Molecular weight standards were thyroglobulin (669-kDa), ferritin (440-kDa), aldolase (158-kDa), conalbumin (75-kDa) and ovalbumin (44-kDa) from the High Molecular Weight Gel Filtration Calibration Kit (GE Healthcare, Little Chalfont, UK). Protein elution volume was monitored by absorbance at 280 nm. All molecular weight estimations were assessed with a standard curve done with the molecular weight standards.

### Non-denaturing gradient PAGE

Novex^™^ 4–20% Tris-Glycine native gels were used for all non-denaturing PAGE assays (Thermo Fisher Scientific, Carlsbad, USA). All samples were diluted in an equal volume of 2X native sample buffer (0.15 M Tris-HCl pH 6.8, 30% glycerol and 0.018 mg/ml bromophenol blue). Migration was performed at 150 volts until the migration front reached the bottom of the gel (~ 2 hours). Proteins were stained with Coomassie Brilliant Blue G-250. NativeMark^™^ (Thermo Fisher Scientific, Carlsbad, USA) was used as protein molecular weight standard.

For oligomeric profiles assessment, 15 μl of 30 μM HspSP-ShM2 and HspS-WH7803 were loaded alone on a native gel and ran at room temperature. When mentioned, sHSP samples were pre-incubated at 45°C during 15 min and electrophoresis was conducted at 45°C. To test for molarity-dependent hetero-oligomerization, different HspSP-ShM2:HspS-WH7803 molar ratios were used: 1:1 (40 μM:40 μM), 2:1 (80 μM:40 μM) and 4:1 (160 μM:40 μM). Samples were incubated 30 min at room temperature prior to loading onto the native gel and electrophoresis was conducted at room temperature. To monitor sHSPs possible heat-stimulated subunit exchange, the two sHSPs were mixed at a 1:1 (40 μM:40 μM) molar ratio and incubated 30 min at 35°C, 45°C or 55°C [6]. The samples were then cooled down 15 min and separated on native gel at room temperature. All samples contained TEN supplemented with 0.12 mM of TritonX-100™.

### Dynamic Light Scattering (DLS)

HspSP-ShM2 was characterized by DLS using a DynaPro instrument (Wyatt Technology Corp., Santa Barbara, CA). The laser light wavelength is 830 nm and the scattered light is collected at 90° by an optic fibre until a detector. All samples were filtered through 0.22 μm filters prior the measurements. Protein concentrations were adjusted from 10 to 100 μM. Sample volumes varied from 20 to 80 μl, the correlation time was 0.5 ms and the acquisition time was 10 s for a total measurement up to 1 h. The sample temperature was controlled with a Peltier cell from 8°C up to 55°C with a precision of 0.1°C. The hydrodynamic radius (Rh) and the standard deviation (polydispersity, %P) were obtained using regularization methods (Dynamics software, version 6) [38, 39].

### In vitro protein client aggregation prevention assays under heat stressed condition

Prevention of heat-induced aggregation assays were done as described in [13–15]. Citrate synthase (CS; 0.1665 μM), malate dehydrogenase (MDH; 0.65 μM) were incubated alone or in presence of HspSP-ShM2 and / or HspS-WH7803 at the given molar ratios for 90 min at 45°C. Luciferase (Luc; 0.1 μM) assays, alone or in presence of sHSPs, were performed at 42°C during only 30 min because of its tendency to reach full aggregation faster than the two other proteins. When mentioned, assay’s buffer was supplemented with 0.12 mM of TritonX-100^™^. Data from light scattering at 320 nm was assessed every minute and are representative of 6 trials. The percentage of aggregation was obtained by dividing each data by the data obtained for the client protein alone at the end of the experiment (90 min for MDH and CS and 30 min for Luc), to which the arbitrary value of 100% of aggregation is attributed. Bovine serum albumin (BSA) and DmHsp27 [15] were used as negative and positive controls respectively. Experiments were done in acrylic semi-micro cuvettes (Sarstedt, Nümbrecht, Germany) in 50 mM HEPES-KOH pH 7.9 buffer supplemented with 0.12 mM TritonX-100^™^ when indicated. Solubility assays were performed as described in [40]. Luc was incubated alone or in presence of HspSP-ShM2 at 6:1, 12:1 and 24:1 Luc:HspSP-ShM2 molar ratios and heated at 42°C for 8 min, while MDH was incubated alone or in the presence of HspSP-ShM2 at 3:1, 6:1 and 12:1 MDH:HspSP-ShM2 molar ratios and heated at 45°C for 60 min. After incubation, samples of both the Luc and MDH assays were centrifuged at 13,000 rpm for 15 min at room temperature to separate the soluble/supernatant and non-soluble/pellet fractions. Soluble fractions were diluted in an equal volume of 2X sample buffer (0.15 M Tris-HCl pH 6.8, 1.2% SDS, 30% glycerol, 5% ß-mercaptoethanol and 0.018 mg/ml bromophenol blue) and pellets were resuspended in 1X sample buffer with the same final volume as the soluble fraction. Equal sample volumes were loaded onto 12% SDS-PAGE and gels were stained with Coomassie Brilliant Blue G-250 following migration.

SEC runs with HspSP-ShM2 and MDH or Luc heated and non-heated were performed as described in the previous section (TEN buffer). HspSP-ShM2 and MDH samples were heated 1 h at 45°C and HspSP-ShM2 and Luc samples were heated 8 min at 42°C, both conditions were cooled down and then centrifuged at 13,000 rpm for 15 min at room temperature prior to load the supernatant onto the column. Protein elution volume was monitored by absorbance at 280 nm.

## Results

### HspSP-ShM2 and HspS-WH7803 display different oligomeric forms depending on protein structure assessment methods

**Fig 1:**
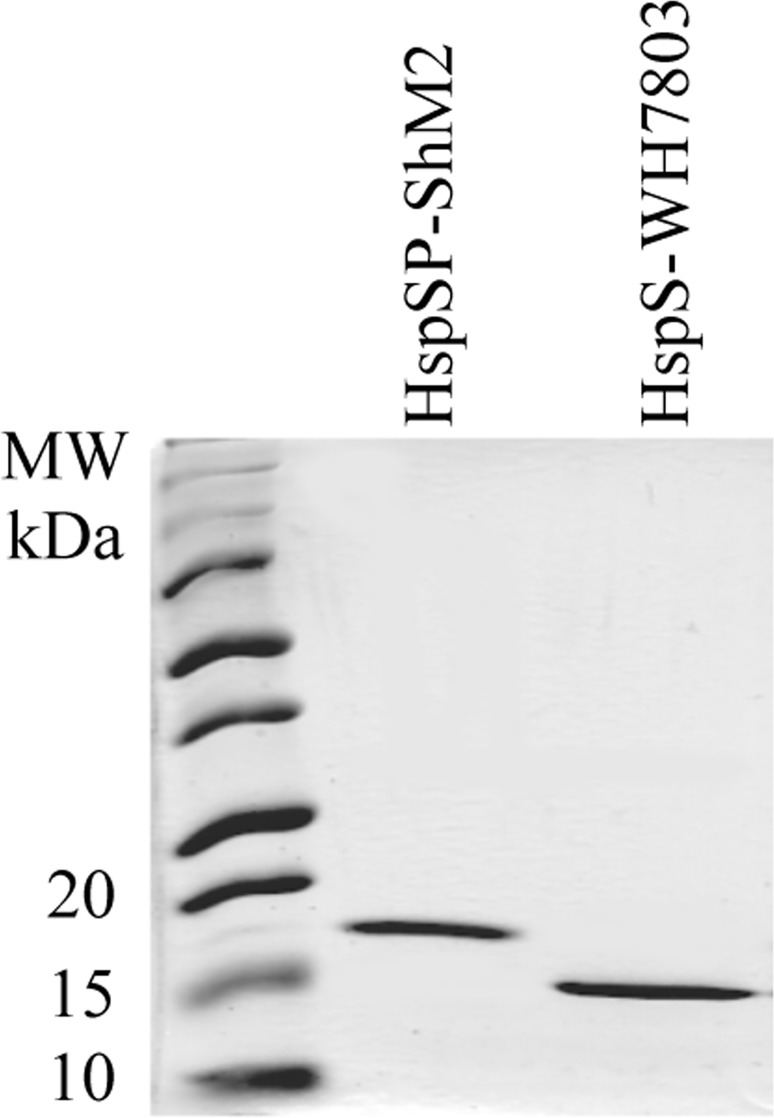
Migration of purified HspSP-ShM2 (18 kDa) and HspS-WH7803 (15 kDa) on SDS-PAGE. 2 μg of purified sHSPs were loaded onto 12% SDS-PAGE to examine purity and MW. The gel was stained with Coomassie Brilliant Blue G-250.

Fig 1 shows the SDS-PAGE gel of purified recombinant cyanophage and cyanobacterial proteins: HspSP-ShM2 migrates at 18 kDa while the sHSP of WH7803 is found at 15 kDa.

**Fig 2:**
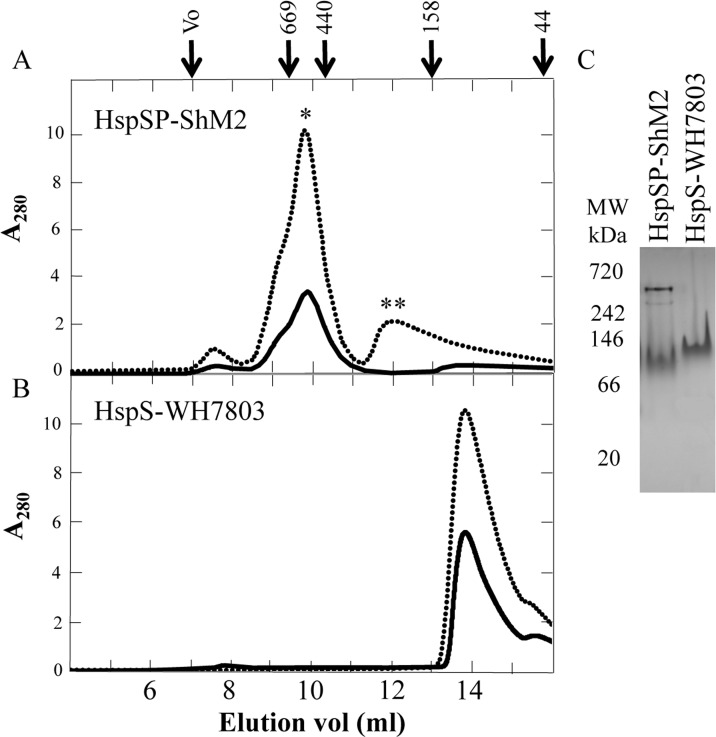
SEC and native gel analysis of HspSP-ShM2 and HspS-WH7803 oligomeric species. Each sHSP was analyzed at two different concentrations, 30 μM (solid line) and 60 μM (dotted line). A) HspSP-ShM2 first peak (*) elution volume is equivalent to ~ 600 kDa (32mer) and the second one (**) to ~ 200 kDa (dodecamer). B) HspS-WH7803 peak elution volume is equivalent to ~ 60 kDa (tetramer). C) Novex™ 4–20% Tris-Glycine native gels were used to compare the oligomeric profiles of HspSP-ShM2 and HspS-WH7803. The elution volumes of protein molecular weight standards are shown in the left panel.

In order to investigate HspSP-ShM2 oligomeric structure, SEC and non-denaturing native gel electrophoresis were used. On SEC HspSP-ShM2 shows a polydisperse oligomeric profile with a main species observed at ~ 600 kDa and a minor one at ~ 200 kDa at the highest concentration used (60 μM) while only the ~ 600 kDa species was observed at 30 μM (Fig 2A). Given the molecular mass of HspSP-ShM2 monomer, which is approximately 18 kDa, the peaks would represent a 32-34mer and a dodecamer respectively. The oligomeric profile of the host cyanobacteria HspS-WH7803 was next examined in the presence of Triton^™^ X-100. On SEC amphitropic HspS-WH7803 (Fig 2B) in 0.23 mM of Triton^™^ X-100 showed an abundant species with an apparent mass of ~ 60 kDa. Considering a 15 kDa monomer, without the Triton micelle, a 60 kDa structure would represent a tetramer, but considering a 3 to 5 nm radius Triton micelle, a 60 kDa structure could be formed by a dimer.

Interestingly, the migration of both sHSPs on native gels under the same buffer and temperature conditions used for the SEC exhibits a different oligomeric profile. Indeed, HspS-WH7803 migration displays a single oligomeric structure as in the SEC assays but it is found at ~ 120 kDa (Fig 2C). In the case of HspSP-ShM2, a shift of the high molecular structures can be seen as well but the most striking observation is the appearance of a third oligomeric state (Fig 2C). Indeed, HspSP-ShM2 complexes are observed at ~ 430 kDa, ~ 325 kDa and ~ 90 kDa on native gels compared to ~ 600 kDa and ~ 200 kDa in the SEC elution profile and the main oligomeric species has shifted from ~ 600 kDa to ~ 90 kDa. Hence, the use of SEC and native gels does not give identical oligomeric size estimates. However, both techniques show that HspSP-ShM2 and HspS-WH7803 form oligomeric structures of different sizes.

In order to discriminate between the HspSP-ShM2 oligomeric profile results, DLS was performed to measure the hydrodynamic radius Rh and the polydispersity degree of the protein assembly. Different temperatures have been tested since differences in temperature and especially high temperatures have proven to stimulate sHSPs conformational changes and subunit exchange resulting in formation of oligomeric species of different sizes [6, 41–43]. At each temperature, the scattered intensity was recorded up to one hour, and the corresponding Rh and the % of polydispersity were calculated.

**Fig 3:**
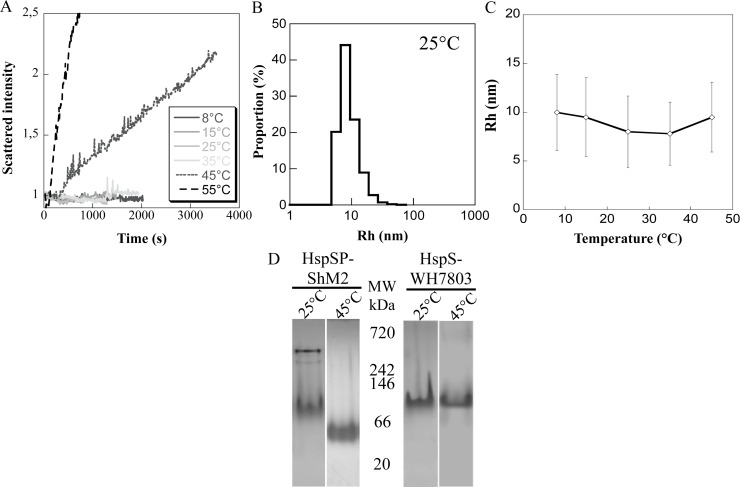
DLS analysis of HspSP-ShM2 oligomers and HspSP-ShM2 but not HspS-WH7803 dissociates into a suboligomeric species when heated. A) Scattered intensity recorded as a function of time for increasing temperatures. B) Proportion of HspSP-ShM2 Rh at 25°C. C) Evolution of Rh with the corresponding standard deviation (polydispersity) at different temperatures. D) To assess the effect of constant heat on structural dynamics, HspSP-ShM2 or HspS-WH7803 were loaded into a 4–20% Tris-Glycine native gel and separated either at 25°C or pre-incubated 15 min and ran at 45°C.

From 8°C to 35°C the scattered intensity (Fig 3A) is constant and each temperature displays a high standard deviation / polydispersity from 39% up to 46%, (monodisperse threshold < 20%) as shown for the assay at 25°C (Fig 3B). This corresponds, at each temperature, to a stable population of several HspSP-ShM2 species with no major conformational or detectable structural transition, such as formation of new species, during the entire hour of data acquisition. However, it is likely that there is subunit exchanges between the oligomeric species, given the structural dynamics features of sHSPs in general. Furthermore, between 8°C and 35°C the Rh of the main oligomeric species, in terms of % of total protein mass, varies from 10.0 nm to 7.6 nm (Fig 3C). According to the globular protein model, an averaged molecular mass was estimated for these species specifically, and decreases from 744 kDa at 8°C, 654 kDa at 15°C, 431 kDa at 25°C to 414 kDa at 35°C. From 45°C, the scattered intensity is no more constant with time and increases strongly as it doubles in one hour (Fig 3A). This is due to the appearance of supplementary HspSP-ShM2 species; hence exhibiting increased structural dynamics especially through large oligomers formation. Together, these new species represent between 15 to 25% of the total protein mass. Meanwhile, the Rh of the main species is about 9.5 nm and the population displays 37% of polydispersity (Fig 3C). In order to assess the effects of such temperature on HspSP-ShM2 through another technique, migration at 45°C in native gels was performed. The HspSP-ShM2 migration profile shows a complete disappearance of the two higher oligomeric species while a single smaller sub-oligomeric species can be observed. Based on molecular mass, this new form could represent a trimer or a dimer (Fig 3D). In contrast, HspS-WH7803 shows no dissociation when heated and migrated at 45°C as shown by the presence of the ~ 120 kDa structural entity (Fig 3D). At 55°C, the very important increase of scattered intensity corresponds to large aggregates formation by HspSP-ShM2.

Although discrepant, the results of the three techniques show that the protein HspSP-ShM2 form large and polydisperse oligomers, whose size is highly dependent on the conditions, especially the protein concentration and the temperature.

### Phage HspSP-ShM2 and bacterial HspS-WH7803 do not co-assemble in vitro

We next examined if the viral sHSP and the one present in the cyanobacteria could interact together since hetero-oligomer formation is a feature observed for some sHSPs when present in the same cellular environment [41, 44–46]. Indeed, infection of *Synechococcus* sp. by SP-ShM2 implies that cyanophage genes are expressed in its bacterial host. Among others, this has been shown for cyanophage genes related to host fitness and this has also been hypothesised for HspSP-ShM2 [29, 33, 36]. Furthermore, structural visualization of both sHSPs using PyMOL software [30] predicted dimers association by docking of the CAM motif from the phage HspSP-ShM2 dimer into β4 and β8 hydrophobic pockets of cyanobacteria HspS-WH7803 dimer and vice versa. Thus, to test if the two sHSPs are capable of forming hetero-oligomers *in vitro*, they were mixed together under several experimental conditions.

**Fig 4:**
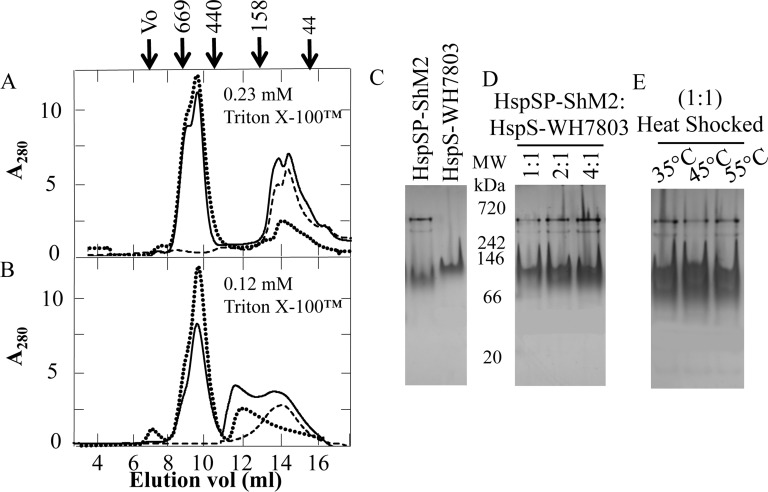
HspSP-ShM2 and HspWH7803 do not form hetero-oligomers at different concentrations of Triton™ X-100, different molar ratios nor under heat treatment. A and B) HspSP-ShM2 (dotted line) and HspS-WH7803 (dashed line) were incubated 30 min either alone or together (solid line) at a molar ratio of 1:1 at two different concentrations of Triton X-100™, (A) 0.23 mM and (B) 0.12 mM. Note that elution peaks of mixed sHSPs conditions for both detergent concentrations are equal as the ones of sHSPs alone. Protein elution volume was monitored by absorbance at 280 nm. The elution volumes of protein molar weight standards are shown above the first panel. C, D and E) Novex™ 4–20% Tris-Glycine native gels were used to compare the oligomeric profiles of (C) HspSP-ShM2 and HspS-WH7803 alone as controls for subsequent comparison (D) HspSP-ShM2 and HspS-WH7803 mixed together at different molar ratios and (E) HspS-ShM2 and HspS-WH7803 mixed together at an equimolar ratio, heat shocked at the indicated temperature, cooled down and migrated at room temperature (~25°C). The migration distances of protein molecular weight standards are shown between the panels C and D.

Therefore, we mixed and incubated both sHSPs at an equimolar ratio in TEN-0.23 mM Triton X-100™ buffer prior to loading onto SEC column. As shown on the graph (Fig 4A) the absorbance peaks match the ones of both HspSP-ShM2 (dotted line) and HspS-WH7803 (dashed line) alone suggesting no apparent interactions and formation of hetero-oligomeric states under these experimental conditions. Interestingly, the CMC (0.23 mM) of Triton X-100™ (Fig 4A, dotted line) displayed no effects on the higher molecular form (~ 600 kDa) of HspSP-ShM2 elution volume but shifted the minor oligomeric species (~ 200 kDa) into a smaller one (~ 68 kDa) with an elution volume of 14.45 ml instead of 12.18 ml. Given the fact that micelles are of amphipathic nature and stably interact with amphitropic proteins by hydrophobic interactions, it is possible that HspS-WH7803 solubilized in TEN-0.23 mM Triton X-100™ buffer is unavailable for interactions with other sHSPs. Thus, to increase HspS-WH7803 availability, both sHSPs were mixed at the same equimolar ratio in TEN-0.12 mM Triton X-100™ buffer. The results (Fig 4B) show a correlation of the absorbance peaks of mixed sHSPs profile with the ones of sHSPs alone, displaying no discernable hetero-oligomeric species. Moreover, HspSP-ShM2 (Fig 4B, dotted line) shows exactly the same elution volume profile as the one for the native experimental condition (Fig 2A) for both oligomeric species. Interestingly, HspS-WH7803 exhibits an oligomeric profile (Fig 4B, dashed line), which is the same as the one observed in the CMC condition SEC assays (Fig 2B) even though the concentration of Triton X-100™ is half the CMC’s. For these reasons and to minimize the effects of non-ionic detergent presence, all further sHSP:sHSP interaction experiments were performed using 0.12 mM of Triton X-100™. To test if the interaction between the two sHSPs could be concentration dependent, three HspSP-ShM2:HspS-WH7803 molar ratios (1:1, 2:1 and 4:1) were tested and analysed using 4–20% native Tris-Glycine gels to compare oligomeric species profiles (Fig 4D). The migration of the 2:1 and 4:1 molar ratios were similar to that observed for the 1:1 ratio exhibiting no conformational or structural changes on native gels. In light of these results, and consistent with the SEC assays, both phage and bacterial sHSPs form stable homo-oligomers when alone or in presence of each other at different concentrations.

Next, a HspSP-ShM2:HspS-WH7803 mixture at equimolar ratio in TEN-0.12 mM Triton X-100™ buffer was heat treated at different temperatures to increase subunit exchange and to stimulate interaction between sHSPs (Fig 4E). Despite the apparent heat-increased subunit dissociation of HspSP-ShM2, no hetero-oligomer was observed between the two sHSPs after heat treatment. Furthermore, all native HspSP-ShM2 oligomeric species seem to reassemble in the cooling process reaching its structural equilibrium at room temperature.

In summary, while both HspSP-ShM2 and HspS-WH7803 may be simultaneously present in the host cyanobacteria cellular environment and even if HspSP-ShM2 and HspS-WH7803 dimers association seems possible *in silico*, no evidence for hetero-oligomer formation was observed under all experimental conditions tested in order to stimulate hetero-oligomer formation.

### HspSP-ShM2 but not HspS-WH7803 successfully prevents the heat-induced aggregation of three different protein clients

**Fig 5:**
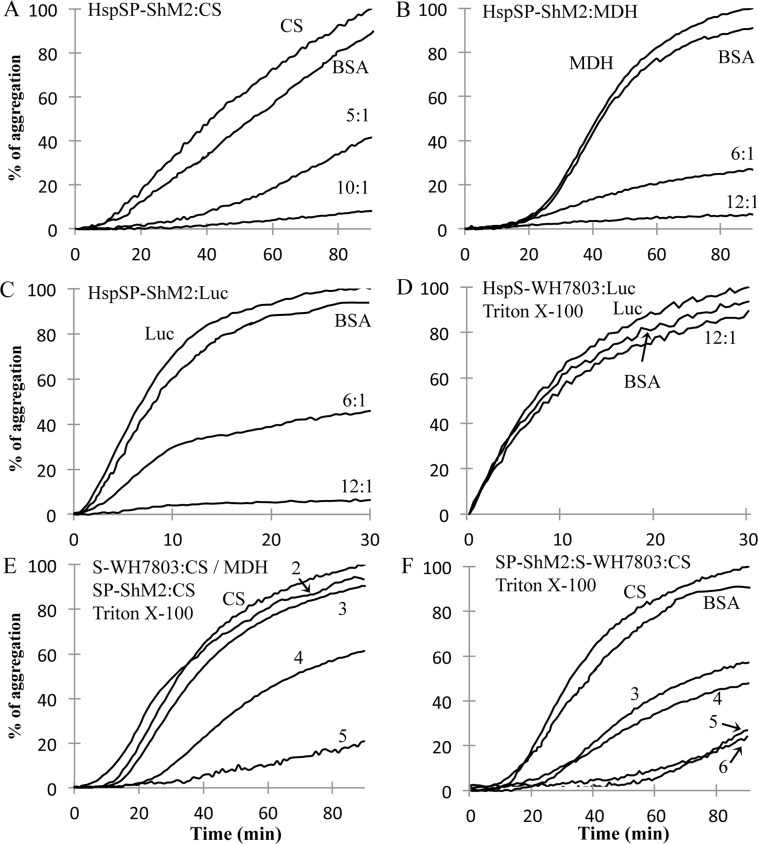
HspSP-ShM2 inhibits heat-induced aggregation of protein clients. A, B and C) CS (A), MDH (B) and Luc (C) were incubated alone or in presence of HspSP-ShM2 at the indicated molar ratios (sHSP:Client). D) Luc was incubated either alone (Luc) or with HspS-WH7803 at the indicated molar ratio. E) MDH was incubated with HspS-WH7803 12:1 (2) and CS was incubated alone (CS), with HspS-WH7803 10:1 (3) or with HspSP-ShM2 at two molar ratios 10:1 (4) and 5:1 (5). F) CS was incubated either alone (CS), in presence of HspSP-ShM2 5:1 (3) and 10:1 (5) or in the presence of HspSP-ShM2 and HspS-WH7803 5:5:1 (4) and 10:10:1 (6).

In addition to oligomer formation in native conditions, preventing irreversible aggregation of proteins under physiological and stressed conditions is another common feature of sHSPs (Reviewed in [2]). Three different clients commonly used in *in vitro* chaperone assays (citrate synthase (CS), malate dehydrogenase (MDH) and luciferase (Luc)) were tested at two different molar ratios for the purpose of characterizing the client-specificity and molar dependent efficiency of the chaperon-like activity of HspSP-ShM2. CS (Fig 5A) shows 41.6±5.7% and 8.17±2.01% of aggregation at 5:1 and 10:1 HspSP-ShM2:CS ratios respectively compared to CS alone. MDH (Fig 5B) aggregation prevention assays show a 26.9±1.9% of MDH aggregation with a 6:1 HspSP-ShM2:MDH molar ratio while the 12:1 ratio displays a 6.5±0.8% of aggregation. Finally, HspSP-ShM2 with 0.2 μM of Luc (Fig 5C) as protein client shows a 45.8±2.2% and 6.4±0.6% of aggregation for the 6:1 and 12:1 HspSP-ShM2:Luc molar ratio respectively. In all the assays, BSA (12:1) was used as a negative control (data not shown in Fig 5E) and showed no significant chaperone like-activity. Similar to HspSP-ShM2, the chaperone-like activity of HspS-WH7803 was investigated using Luc at a molar ratio of 12:1 (Fig 5D), MDH at a molar ratio of 12:1 (Fig 5E, curve #2) and CS at a molar ratio of 10:1 (Fig 5D, curve #3) (HspS-WH7803:client). Surprisingly no significant chaperone-like activity was observed in any of the assays performed, as shown in Luc, MDH and CS assays with percentages of aggregation of 86.7±6.4%, 90.6±3.7% and 85.8±5.5% respectively.

To test if the presence of Triton X-100™ CMC could account for the lack of HspS-WH7803 chaperone-like activity, prevention of heat-induced aggregation assays in presence of 0.23 mM Triton X-100™ were done with HspSP-ShM2 (Fig 5E, curve #4 and #5) and *Drosophila melanogaster* Hsp22 and Hsp27 known for their chaperon-like activity in these assays [15]. HspSP-ShM2 showed a decreased ability of 20% and 12% in preventing aggregation of CS at 5:1 and 10:1 (HspSP-ShM2:CS) molar ratios respectively (compare Fig 5A with Fig 5E, curves #4 (5:1) and #5 (10:1)). For both DmHsp22 and DmHsp27, on the other hand, no changes in chaperon like activity were noted in presence of Triton X-100™ (data not shown). Consequently, the presence of non-ionic detergent can partially impairs the ability of some but not all sHSPs to assemble with clients in the process of aggregation by recognition of hydrophobic exposed surfaces. Therefore, Triton X-100™ CMC doesn’t seem to be entirely responsible for the lack of HspS-WH7803 chaperone activity.

Despite the lack of HspS-WH7803 *in vitro* chaperon-like activity or association with cyanophage sHSP, heat-induced aggregation prevention assays with both sHSPs together have been performed in order to search for any functional cooperation or repression between HspSP-ShM2 and HspS-WH7803 (Fig 5F). HspSP-ShM2, HspS-WH7803 and CS were mixed at a molar ratio of 5:5:1 (curve #4) and 10:10:1 (curve #6) and compared to HspSP-ShM2 alone with CS at the same molar ratios (5:1, curve #3) and (10:1, curve #5). In both assays, there are no significant changes in substrates aggregation, suggesting no mutual effects of both sHSPs regarding their chaperon-like activity.

In summary, these results show an ability of HspSP-ShM2, but not HspS-WH7803, to prevent the aggregation of different protein clients in thermal assays and show no functional alterations when both HspSP-ShM2 and HspS-WH7803 are present together.

### HspSP-ShM2 prevents client’s aggregation by preserving their soluble state via formation of high molecular weight sHSPs:clients complexes

**Fig 6:**
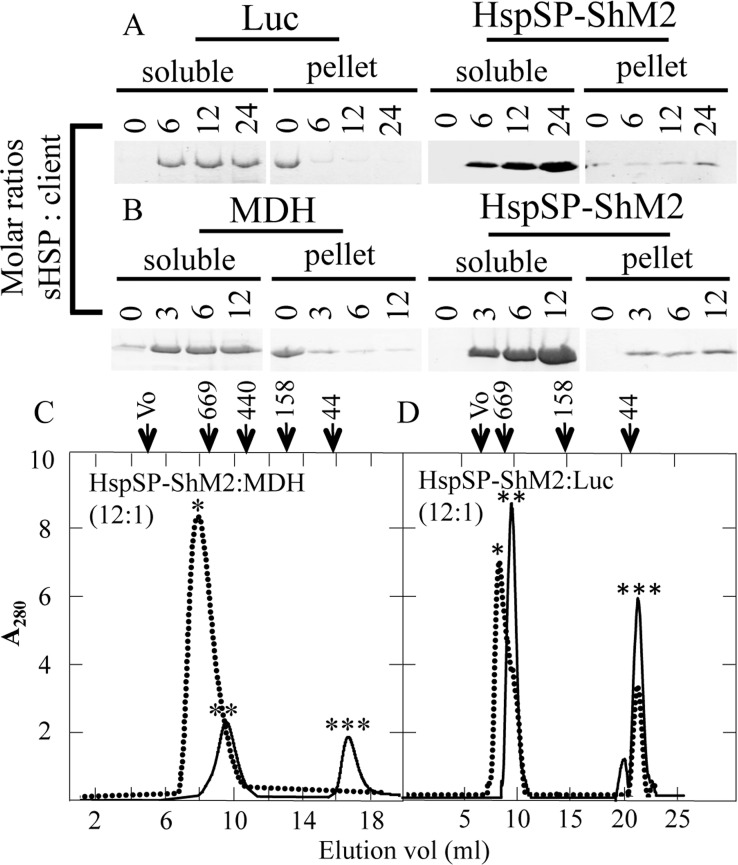
HspSP-ShM2 maintains heat stressed protein clients soluble and form stable complexes. A and B) Luc and MDH clients were incubated either alone or in presence of HspS-ShM2 at the indicated molar ratios. Both Luc and MDH fully aggregate in absence of HspSP-ShM2 as shown by their presence in the pellet fraction while both are maintained soluble when in presence of HspSP-ShM2. C) MDH and HspSP-ShM2 were incubated for 60 min at 45°C (dotted line) or at room temperature (solid line) at a 12:1 (sHSP:MDH) molar ratio and D) Luc and HspSP-ShM2 were incubated for 8 min at 42°C (dotted line) or at room temperature (solid line) at a 12:1 (sHSP:Luc) molar ratio. Note that HspSP-ShM2 forms complexes with MDH and Luc when heated (dotted line) since the peaks (*) have a lower elution volume, therefore representing a higher molecular weight than HspSP-ShM2 alone (**). Moreover, the peak representing MDH when not heated (C) (***) completely disappears after heat treatment. The elution volumes of protein molecular weight standards are shown above the panel.

Following the *in vitro* validation of HspSP-ShM2 ability to prevent protein aggregation, other tests were carried out in order to determine if this chaperone-like activity is the result of formation of soluble HspSP-ShM2-clients complexes after heat shock, consistent with the functional properties of oligomeric sHSPs in general [20, 40, 47]. Therefore, it was of interest to establish a correlation between the aggregation prevention activity of HspSP-ShM2 and the preservation of client solubility under heat stress. The clients and sHSP for all ratios tested were practically all recovered in the soluble fraction while the clients alone were recovered in the insoluble pellet (Fig 6A and 6B). These results provide additional evidence on the ability of HspSP-ShM2 to prevent protein insolubilization/aggregation and, therefore, confirm its *in vitro* chaperone like-activity. Thus, to examine the possible interaction and complex formation between HspSP-ShM2 and client, SEC analysis were performed with heated and non-heated HspSP-ShM2-MDH and HspSP-ShM2-Luc samples at a 12:1 (sHSP: client) molar ratio whereby HspSP-ShM2 fully protects MDH and Luc from aggregation as observed in the aggregation prevention and solubility experiments (Fig 5B and 5C). In the absence of heat treatment (Fig 6C and 6D, solid lines), HspSP-ShM2, MDH and Luc eluted separately at their predicted molecular mass implying no interaction between the sHSP and its client as expected. Once heated (Fig 6C and 6D, dotted line) the samples supernatants SEC analysis displays an elution peak of greater molecular mass then HspSP-ShM2 alone in native condition representing complexes of denatured MDH and Luc in association with HspSP-ShM2. A complete disappearance of the sHSP and MDH elution peaks was also observed in the heated condition suggesting their complete association in this experimental condition.

Taken together, the overt structural dynamics of HspSP-ShM2 at higher temperature and its chaperone-like activity suggest the dissociation of HspSP-ShM2 subunits into smaller oligomeric species under stress conditions, which then bind denatured client and form stable complexes of high molecular weight with the client in order to prevent its irreversible aggregation.

## Discussion

Cyanophage HspSP-ShM2 was shown to form two main oligomers by SEC, the latter species seeming to be concentration dependent, whereas native gels electrophoresis and DLS displayed an overall shift of oligomeric structures and a more polydisperse profile. The sHSP concentration was the main changing factor between the different experiments since the buffer and temperature conditions were the same. Therefore, along with the choice of method employed, this could explain the differences of oligomeric species observed between the techniques. Indeed, the use of different methods to assess protein structural dynamic and identification of structural entities has proven to reveal different results depending on the sensitivity and measurement methodology of the experimental technique. This was also noted for *Saccharomyces cerevisae* Hsp26. In fact SEC analysis of ScHsp26 exhibited only one oligomeric structure portraying the protein as one highly abundant species [43], while mass spectrometry on the other hand was able to detect a wide range of structures ranging from monomers to 40-mers [48]. Thus, the different HspSP-ShM2 oligomeric species observed at equilibrium with native gels and FPLC and the apparent polydispersity at each temperature observed by virtue of DLS suggest a high structural dynamism and oligomeric heterogeneity resulting from rapid subunits exchange differently detected by SEC, native gels and DLS. In addition to sHSPs oligomeric behaviour influenced by physico-chemical factors, variations within the *in vivo* cellular environment and fluctuations met by the virus and host cyanobacteria *Synechococcus* sp. WH7803 might also influence the oligomeric dynamics of HspSP-ShM2 or stimulate the formation of different oligomeric forms. Nevertheless, the capacity of HspSP-ShM2 to form high molecular weight oligomers and to display high structural heterogeneity and dynamics in response of changing conditions and monomer availability are interesting features consistent with those of sHSPs in general.

Since oligomerization and structural dynamics are proposed to be two important aspects for chaperone function [49–51], the HspSP-ShM2 structural behaviour described above was relevant enough for chaperone-like activity investigation. In fact, several observations support the paradigm for which smaller species, especially dimers, are the sub-oligomeric active states for client binding following stress-induced oligomer dissociation [3, 12, 52]. Thus, under constant heat as a stress and enhancing factor for subunits exchange, native gels electrophoresis and DLS have clearly demonstrated the impact of temperature increase on structural dynamics. At 45°C, the subunits could adopt a partially « open / expanded » conformation resulting in the weakening of intra-subunit and inter-subunit interactions in order to release the client binding sites, possibly explaining, in addition to large oligomers formation, the increase of the Rh [48, 53, 54]. Also, it has been proposed that such quaternary conformational rearrangement is a prerequisite for oligomer dissociation and seems to be the case for HspSP-ShM2 since its migration at 45°C on native gel showed the dissociation of the native oligomers into what appears to be dimers or trimers, representing potential heat-stressed active suboligomeric species. Note that when HspSP-ShM2 was heat shocked and, then cooled down it was able to reform its structural pattern when at equilibrium in native conditions, highlighting the high degree of plasticity behaviour of sHSPs in general. Nevertheless, this heat-induced structural dynamism of HspSP-ShM2 was positively translated into an ATP-independent chaperone-like activity dependent on the nature and stoichiometry of the client. More precisely, protection was achieved through the formation of homogeneous high molecular HspSP-ShM2-client soluble complexes with higher molecular weight than native HspSP-ShM2 oligomeric species as shown with MDH and Luc. Importantly, no sHSP-MDH nor sHSP-Luc complexes formation was observed in absence of protein aggregation-induced stresses, which indicates the necessity of client to enter into the process of denaturation and hydrophobic surface exposure for HspSP-ShM2 recognition. However, direct establishment of HspSP-ShM2 sub-oligomers as active binding species needs further proof. Overall these *in vitro* features of HspSP-ShM2 chaperone-like activity go along with the present model described for sHSPs (Reviewed in [2, 55]).

In the *in vivo* context HspSP-ShM2 might as well exert its chaperone-like activity for several host clients participating in many cellular processes since it has been proven to be a promiscuous chaperone *in vitro* as demonstrated for *Synechocystis* PC 6803 Hsp16.6 [18]. Indeed, Basha and Vierling first demonstrated Hsp16.6 ability to prevent Luc from aggregation in a *in vitro* assay which translated into protection of up to 42 proteins in a *in vivo* and heat stressed context. Protected proteins by virtue of Hsp16.6 action were positively refolded through ATP-dependent chaperones and mainly involved in cyanobacteria secondary metabolism, gene transcription, protein translation, photosynthesis and intracellular signalling displaying a wide range of chaperone related functions. Such targets for HspSP-ShM2 would have it tightly involved in host metabolism and ability to cope with stress, indirectly impacting phage faith through the promotion of host survival. Alternatively, HspSP-ShM2 could be involved in chaperoning viral structural proteins especially in regard of co-expression with other viral proteins. HspSP-ShM2 could conduct the correct folding of viral proteins while undergoing pre-lysis massive protein production. It could act independently and hold misfolded viral proteins for self-promoted refolding. Alternatively, it could act in cooperation with cyanobacterial or cyanophage encoded ATP-dependent chaperone machinery for active folding and virus production. Indeed, some bacteriophages are known to possess an ATP-dependent machinery for protein folding. More precisely, T4 [56] and RB49 [57] bacteriophages possess a gene encoding for a co-chaperonin ortholog of bacterial GroES, namely gp31 and CocO respectively, while the presence of a GroEL chaperonin gene ortholog has been confirmed in several phage genomes [58, 59]. Further investigations from Kurochikna et al. [60] and Semenyuk et al [59, 61] have demonstrated and characterized the GroEL orthologs ATP-dependent chaperon activity of *Pseudomonas aeruginosa* EL and *Pseudomonas fluorescens* OBP bacteriophages. Such genes could be present in cyanophage genomes and protein production could allow investigating for a functional cooperation between phage’s sHSP, chaperonin (GroEL) and / or co-chaperonin (GroES) orthologs. More generally, classical client refolding assays in presence of ATP-dependent chaperone machinery could give proof of HspSP-ShM2’s ability to cooperate with cellular components for active protein refolding and hints for its actual *in vivo* efficiency and functional behaviour. Still, we can suggest that HspSP-ShM2 acts as a classical and functional sHSP based on the *in vitro* characteristics exhibited in the present experiments. Given the duality of phage sHSP evolution history and putative functional implications of cyanobacteria derived phage genes, phage sHSP could be involved in biological functions for viral and / or cyanobacteria metabolism and survival with direct or indirect repercussion on viral cycle.

Concerning HspS-WH7803, the necessity to use detergent micelles to avoid its complete aggregation following tag removal underlies the amphitropic nature of cyanobacteria sHSPs and is in agreement with their role in membrane fluidity regulation [24, 26, 27, 62–64]. HspS-WH7803 forms a small structural entity in presence of Triton^™^ X-100 as shown by SEC (60 kDa) and native gel (100 kDa), which is consistent with the possible anchoring with membranes. Indeed membrane-anchored sHSP species seem usually small since high molecular oligomers might disrupt membrane integrity leading to cell death. For example, *Mycobacterium tuberculosis* Hsp16.3 oligomers [65] need to dissociate into smaller species and especially nonamers for plasma membrane association. Interestingly, the association rate was increased in presence of *M*. *tuberculosis* specific membrane lipids. This observation points toward the involvement of species specific membrane-associated effectors in regards of proper structural dynamics, lipid association and solubility. Moreover, Nitta and colleagues [26] have demonstrated the changes in HspA subcellular localisation in *Synechococcus elongatus* strain PCC 7942 under physiological and heat shock conditions going from cytoplasmic to thylakoid areas and vice versa, which implies at some point solubility of HspA unrelated to amphipathic structures. Among cyanobacteria, the « heat shock lipid » monoglucosyldiacylglycerol (MGlcDG) selectively interacts with HspA to regulate membrane fluidity under stress conditions [24] and could be required for HspS-WH7803 turnover between the functional oligomeric lipid-free and lipid-bound states. Therefore, based on this observation and the need of Triton^™^ X-100 for HspS-WH7803 structural stabilisation, the effect of HspSP-ShM2 presence on HspS-WH7803 solubility through hetero-oligomerization was investigated. Unfortunately, none of the conditions tested and techniques used has permitted us to validate a possible interaction between HspS-WH7803 and HspSP-ShM2. However, based on previous studies [18, 66, 67] and since conformational stability of HspS-WH7803 could be reached, the inability of the sHSP to display any chaperone-like activity in classical aggregation prevention assays was surprising. The reason for the lack of HspS-WH7803 chaperone activity is not known. However, it could be due to stable hydrophobic interactions with the Triton^™^ X-100 micelles leading to its inability to behave and fold correctly into tertiary functional conformation as supported by HspS-WH7803 lack of structural dynamics at higher temperature (45°C). *In vivo* observations of cyanobacteria species overexpressing tagged or untagged HspA have shown an enhanced acclimation to several stresses and stress response through proteostasis maintenance [18, 66, 67]. Such observations indicate that cyanobacteria sHSPs usually possess a functional chaperone activity. Thereby, all these observations reflect the need of working in the *in vivo* cellular environment or with at least species-specific cellular and / or molecular components for further structural and functional studies of native cyanobacteria HspS-WH7803.

To our knowledge this is the first time that a phage sHSP is purified and characterized *in vitro*. In the light of these results, HspSP-ShM2 displays canonical sHSP *in vitro* features as revealed by its chaperone-like activity, oligomerization and structural dynamics while behaving differently than its host counterpart. Further *in vivo* experiments should provide a better portrait of HspSP-ShM2 actual expression, functional impact and relationship with its host. Nonetheless, modular evolution theory [68] whereby viruses evolutionary path is one of functional genes acquisition from diverse sources for a better « fitness » strongly suggest *in vivo* expression of functional HspSP-ShM2. Whether it is implicated in viral maturation and / or host survival and metabolism, HspSP-ShM2 very likely has an overall significant impact considering cyanophage abundance in oceans and their importance on planktonic communities turnover and, therefore, biogeochemical cycles, aquatic and terrestrial food chains [69–71].
